# Interactions between ShPP2-1, an F-box family gene, and ACR11A regulate cold tolerance of tomato

**DOI:** 10.1038/s41438-021-00582-3

**Published:** 2021-07-01

**Authors:** Jianwen Song, Lele Shang, Shiwei Chen, Yongen Lu, Yuyang Zhang, Bo Ouyang, Zhibiao Ye, Junhong Zhang

**Affiliations:** Key Laboratory of Horticultural Plant Biology (MOE) and National Center for Vegetable Improvement (Central China), Huazhong Agricultural University, Wuhan, 430070 China

**Keywords:** Abiotic, Plant physiology

## Abstract

There is a critical need to identify germplasm resources and genes that promote cold tolerance of tomato because global tomato production is threatened by cold stress. We found that the expression of an F-box gene family member named *ShPP2-1* from *Solanum habrochaites* is cold inducible and studied its contribution to cold tolerance. Overexpression of *ShPP2-1* in cultivated tomato (AC) reduced cold tolerance by intensifying damage to cell membranes. To explore the underlying molecular mechanism, we conducted a yeast two-hybrid library screen and found that a protein containing ACT domain repeats named ACR11A interacts with PP2-1. Overexpression of *SlACR11A* in AC enhanced the cold tolerance of seedlings and germinating seeds. Cold tolerance decreased in tomato plants that overexpressed both of these genes. Additionally, we performed seed germination experiments in the cold with 177 tomato accessions and identified two alleles of *SlACR11A* that differ in one single-nucleotide polymorphism. We found that one of these alleles, *SlACR11A*^*G*^, is significantly enriched in cold-tolerant tomato plants. Taken together, our findings indicate that the combination of low expression levels of *PP2-1* and high expression levels of *ACR11A* can promote cold tolerance. These genes may therefore serve as direct targets for both genetic engineering and improvement projects that aim to enhance the cold tolerance of tomato.

## Introduction

Tomato (*Solanum lycopersicum*) originated in the Andes region of South America, and it was domesticated in Central America and has since been distributed worldwide. As a horticultural crop species with a tropical origin, tomato grows well under warm temperatures. Cold stress is a major environmental stress that severely attenuates the vegetative and reproductive growth of tomato plants and thus restricts the geographic distribution of tomato. Indeed, cold stress causes tremendous losses of tomato crops every year^1^. Plants have evolved complex mechanisms that help them sense, respond, and adapt to cold stress. To breed modern cold-tolerant varieties of tomato, it is essential for us to exploit resistant germplasm resources and to further understand the physiological and biochemical processes that promote cold tolerance.

Previous studies have thoroughly elucidated complex cold-responsive signaling mechanisms in *Arabidopsis thaliana*^2–4^. However, tomato and Arabidopsis respond differently to cold stress (e.g., tomato does not acclimate to cold)^5^. The genetic variation and regulatory mechanisms that promote cold tolerance of tomato thus remain poorly understood. Generally, the indicators used to assess cold tolerance of plants include seed germination, seedling survival and growth, vegetative growth, and reproduction^6^. For instance, by the use of near-isogenic lines (NILs), *COLD1* was found to promote chilling tolerance in rice seedlings^7^. In tomato, particular genes have been found to regulate the cold tolerance of seedlings^8–10^. However, major gaps remain in our knowledge of cold tolerance of tomato. Moreover, a number of quantitative trait loci (QTLs) for cold tolerance during seed germination were identified via backcrosses and the use of recombinant inbred lines (RILs)^11–14^. Until now, none of them have been functionally validated in tomato.

The F-box protein family is one of the largest protein families in plants, and its members are broadly involved in secondary metabolism, phytohormone signaling, plant developmental processes, and stress responses^15^. F-box proteins are characterized by conserved F-box motifs, which contain approximately 40 to 60 amino acid residues and are located at the N-terminus^16^. Based on a secondary motif in the C-terminal region, F-box proteins are classified into several subfamilies that include leucine-rich repeat (LRR), kelch repeat, phloem protein 2 (PP2), F-box associated (FBA), TUBBY (TUB), and WD-40 proteins^15^. PP2 is one of the most mysterious and abundant proteins in plant phloem. Based on the conserved motifs in the PP2 domain and N-terminal region, Arabidopsis PP2 proteins can be divided into two subgroups—PP2-A and PP2-B. Each subgroup contains 15 members^17^. A previous study demonstrated that an F-box protein named AtPP2-B11 negatively regulates drought stress and ABA signaling by binding LEA14 and SnRK2, respectively^18,19^. However, *AtPP2-B11* was also demonstrated to promote salt tolerance by inhibiting the accumulation of reactive oxygen species and by influencing the balance of Na^+^^20^. Moreover, phylogenetic analyses showed that members of the PP2 family are common in angiosperms^17^. Although tomato contains numerous PP2 proteins, our knowledge of PP2 proteins in tomato is rather limited, especially our knowledge of their contribution to cold tolerance.

The ACT domain was named for its presence in bacterial aspartate kinase (AK), chorismate mutase (CM), and prephenate dehydrogenase (PDH and TyrA). The ACT domains of these enzymes serve as amino acid-binding sites that contribute to the allosteric regulation that regulates the flux through amino acid biosynthetic pathways^21,22^. Members of an ACT domain repeat (ACR) protein family have been identified in plants. According to amino acid sequence alignments, there are 12 ACR proteins in Arabidopsis^23,24^. Based on the number of ACT domains and a phylogenetic analysis, the 12 ACR proteins can be divided into three groups. The proteins in Group I, Group II, and Group III—ACR1 to ACR8, ACR9 and ACR10, ACR11 and ACR12—contain four, three, and two copies of the ACT domain, respectively^24^. In Arabidopsis, *ACR11* was shown to modulate defense responses and disease resistance by influencing the accumulation of ROS and SA^25^. Other *ACR* genes have not been cloned or functionally analyzed in either Arabidopsis or tomato.

Our previous study showed that the expression of an F-box gene, *PP2-1*, was strongly induced by cold stress in a cold-tolerant accession compared with a cold-sensitive accession, which suggests that *PP2-1* may play an essential role in the response to cold stress in tomato^26^. However, the function of this gene underlying cold tolerance has not been further studied. Here, we identified and confirmed that the expression of *ShPP2-1* is cold inducible and that *ShPP2-1* is differentially expressed in cold-tolerant *Solanum habrochaites* and cold-sensitive *S. lycopersicum*. We found that the overexpression of *ShPP2-1* decreased cold tolerance by interacting with *SlACR11A* in tomato. In addition, we identified two allelic variants of *SlACR11A* and found that the *SlACR11A*^*G*^ allele is significantly enriched in cold-tolerant tomato accessions. Our research provides new insight into the biological function of *ShPP2-1* and reveals a new molecular mechanism that contributes to cold tolerance of tomato.

## Results

### Response of *PP2-1* expression to different stresses

**Fig. 1:**
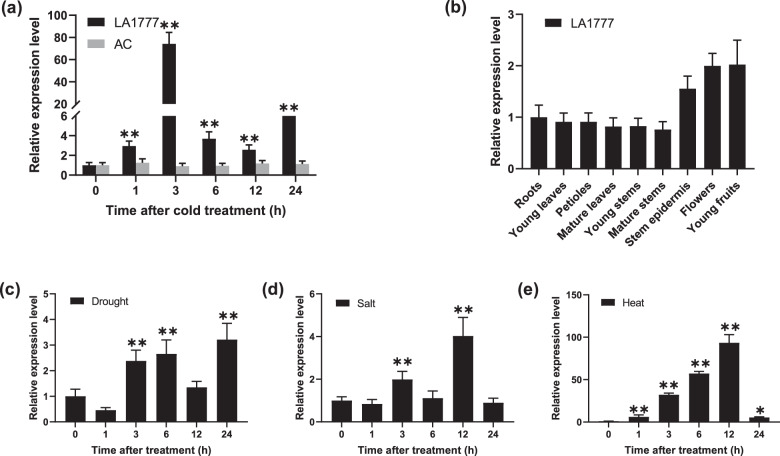
Expression patterns of *PP2-1* in different tissues and in response to particular types of abiotic stress. **a** Expression levels of *PP2-1* in leaves of cold-stressed *S. habrochaites* (LA1777) and *S. lycopersicum* (AC). **b** Expression patterns of *ShPP2-1* in different tissues collected from four-week-old seedlings of LA1777. **c**–**e** Expression pattern of *ShPP2-1* in drought-, salt-, and heat-stressed LA1777 seedlings. For **a** and **c**, four-week-old seedlings were stressed with cold (4 °C), drought (dehydration), salt (200 mM NaCl), or heat (40 °C °C) treatments for 0, 1, 3, 6, 12, or 24 h, respectively. The relative expression levels were determined using qRT-PCR. The expression levels of the appropriate controls were set to a value of 1. Three biological replicates were analyzed for each of the indicated lines. Each replicate was composed of leaves of three seedlings. The bars indicate the mean values. The error bars indicate the SEs. The asterisks indicate statistically significant differences relative to the appropriate control according to Student’s *t* test (*P < 0.05; **P < 0.01)

In our previous microarray analysis, we found that the expression of an F-box gene, *PP2-1*, was strongly induced by cold stress in two cold-tolerant accessions of tomato, LA1777 (*S. habrochaites*) and LA3969 (*S. lycopersicum*), relative to a cold-sensitive accession, LA4024 (*S. lycopersicum*)^26^. To independently evaluate our microarray results, the transcript levels of *PP2-1* were analyzed in LA1777 and AC (a cold-sensitive accession) that were subjected to cold stress. The expression level of *ShPP2-1* significantly increased in LA1777 after cold treatment, especially after a 3-h cold treatment (Fig. 1a). However, cold treatment had no effect on the expression level of *SlPP2-1* in AC (Fig. 1a). These data indicate that the expression of *ShPP2-1* is cold inducible in LA1777, which is consistent with our previous microarray results. To test whether *ShPP2-1* might contribute to responses to other abiotic stresses, we examined the expression pattern of *ShPP2-1* in LA1777 plants after subjecting them to drought, salinity, and heat stress (Fig. 1c–e). The expression of *ShPP2-1* was strongly induced by heat treatment, and a mild induction of *ShPP2-1* expression was detected in response to drought and salt stress. We also analyzed the expression pattern of *ShPP2-1* in different tissues of LA1777 plants that were grown under optimal conditions and found that *ShPP2-1* was expressed in all the tissues tested (Fig. 1b).

### Overexpression of *ShPP2-1* reduced the cold tolerance of tomato

**Fig. 2:**
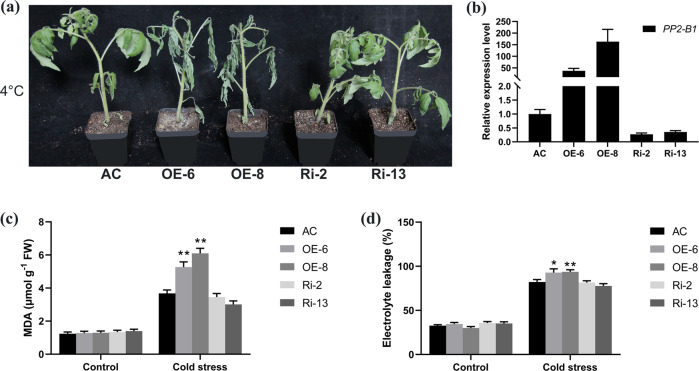
Reduced cold tolerance of the *ShPP2-1* overexpression lines. **a** Phenotypes of transgenic seedlings expressing abnormal levels of *ShPP2-1* and wild-type (AC) seedlings under cold-stress conditions. The overexpression lines (OE-6 and OE-8), RNAi lines (Ri-2 and Ri-13), and AC were subjected to cold stress. Four-week-old transgenic T_2_ and AC seedlings were grown at 4 °C for 6 days during the winter. **b** Relative expression levels of *PP2-1* in transgenic lines and AC. The expression levels of the control (AC) were set to a value of 1. Comparison of MDA contents (**c**) and relative electrolyte leakage (**d**) in the leaves of transgenic lines expressing abnormal levels of *PP2-1* and AC grown under cold stress and optimal conditions. Three biological replicates were analyzed for each line under each condition. Each replicate was composed of leaves of three different seedlings. The bars indicate the mean values. The error bars indicate the SEs. The asterisks indicate statistically significant differences relative to the wild type grown under the same conditions according to Student’s *t* test (*P < 0.05; **P < 0.01)

To characterize the biological function of *ShPP2-1* in tomato, a 35 S:*ShPP2-1* overexpression construct was introduced into AC. Eighteen stable overexpression lines were obtained. Fifteen stable AC RNAi lines were also generated in which the expression of *PP2-1* was knocked down. Two overexpression lines (OE-6 and OE-8) and two RNAi lines (Ri-2 and Ri-13) expressing abnormal levels of *PP2-1* were selected for functional analysis (Fig. 2b).

Four-week-old T_2_ and AC seedlings were chosen and subjected to cold stress (4 °C). After 6 days of cold stress, the *ShPP2-1*-overexpression lines were severely wilted. In contrast, wilting was much less severe for the RNAi lines and AC (Fig. 2a). The levels of MDA and relative electrolyte leakage reflect the integrity of cellular membranes, and MDA production after cold stress can be used as a lipid peroxidation marker^27^. Therefore, we quantified the MDA and relative electrolyte leakage levels in the cold-treated plants. Under control conditions, there were no differences in the levels of MDA or relative electrolyte leakage in the transgenic and AC plants (Fig. 2c, d). After 6 days of cold treatment (4 °C), the MDA and relative electrolyte leakage levels increased in both the transgenic and AC plants. However, the levels in the overexpression lines were significantly higher than those in the RNAi lines and wild-type AC (Fig. 2c, d). Our results demonstrate that the damage to cellular membranes that occurs during cold stress increased in plants that overexpress *ShPP2-1*. Based on these data, we concluded that *ShPP2-1* negatively regulates cold tolerance by intensifying cell membrane damage in tomato plants that experience cold stress.

### ShPP2-1 interacts with ACR11A, an ACR protein

**Fig. 3:**
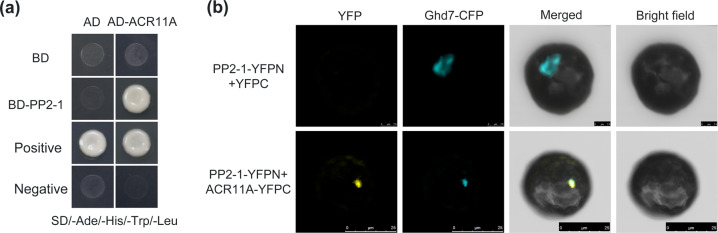
Interactions between PP2-1 and ACR11A. **a** Analysis of interactions between PP2-1 and ACR11A in yeast. Recombinant plasmids containing either pGBKT7-53 and pGADT7-RecT or pGBKT7-Lam and pGADT7-RecT were introduced into yeast AH109 cells and used as positive and negative controls, respectively. Yeast cells were cultured on SD/-Trp/-Leu/-His/-Ade media. **b** Interactions between PP2-1 and ACR11A in *N. benthamiana* protoplasts. With the use of PEG-based method, *N. benthamiana* protoplasts were cotransformed with PP2-1-YFPN and ACR11A-YFPC or with a YFPC empty vector. Interactions were detected using BiFC assays. YFP fluorescence is indicated in yellow. Fluorescence of the nuclear marker is indicated in blue. The nuclear marker Ghd7-CFP specifically accumulates in the nuclei of rice cells

To determine the role of *ShPP2-1* in cold tolerance, we conducted a yeast two-hybrid screen and identified 14 candidate interactors (Table S2). Among them, ACR11A was selected for further analysis because it was previously reported to modulate defense responses and disease resistance^25^. We cotransformed pGBKT7-ShPP2-1 and pGADT7-ACR11A in yeast cells and found that the transformants grew on SD/-Ade/-His/-Leu/-Trp media, the results of which were consistent with those of the positive control yeast cells (Fig. 3a), indicating that ShPP2-1 interacts with ACR11A in yeast. To further verify this interaction, we performed bimolecular fluorescence complementation (BiFC) assays. We transiently co-overexpressed YFPN-tagged ShPP2-1 and YFPC-tagged SlACR11A in *Nicotiana benthamiana* leaf protoplasts and observed that YFP fluorescence signals colocalized together with the nuclear marker (Fig. 3b). Taken together, these data demonstrated that ShPP2-1 interacts with SlACR11A.

### *SlACR11A* positively regulates the cold tolerance of tomato

We identified 14 *ACR* genes in the tomato genome with a BLAST search and a phylogenetic analysis: *SlACR1* to *SlACR13* (Fig. S1). We then divided the tomato *ACR* genes into 3 groups (I, II, and III based on whether they encode proteins that contain four, three, or two copies of the ACT domain, respectively. In contrast to findings in Arabidopsis, there were 9 members in Group I (*SlACR1*-*SlACR8* and *SlACR13*), 2 members in Group II (*SlACR9* and *SlACR10*), and 3 members in Group III (*SlACR11A*, *SlACR11B*, and *SlACR12*) (Fig. S1). *SlACR11A* and *AtACR11* were in the same clade (Fig. S1).

**Fig. 4:**
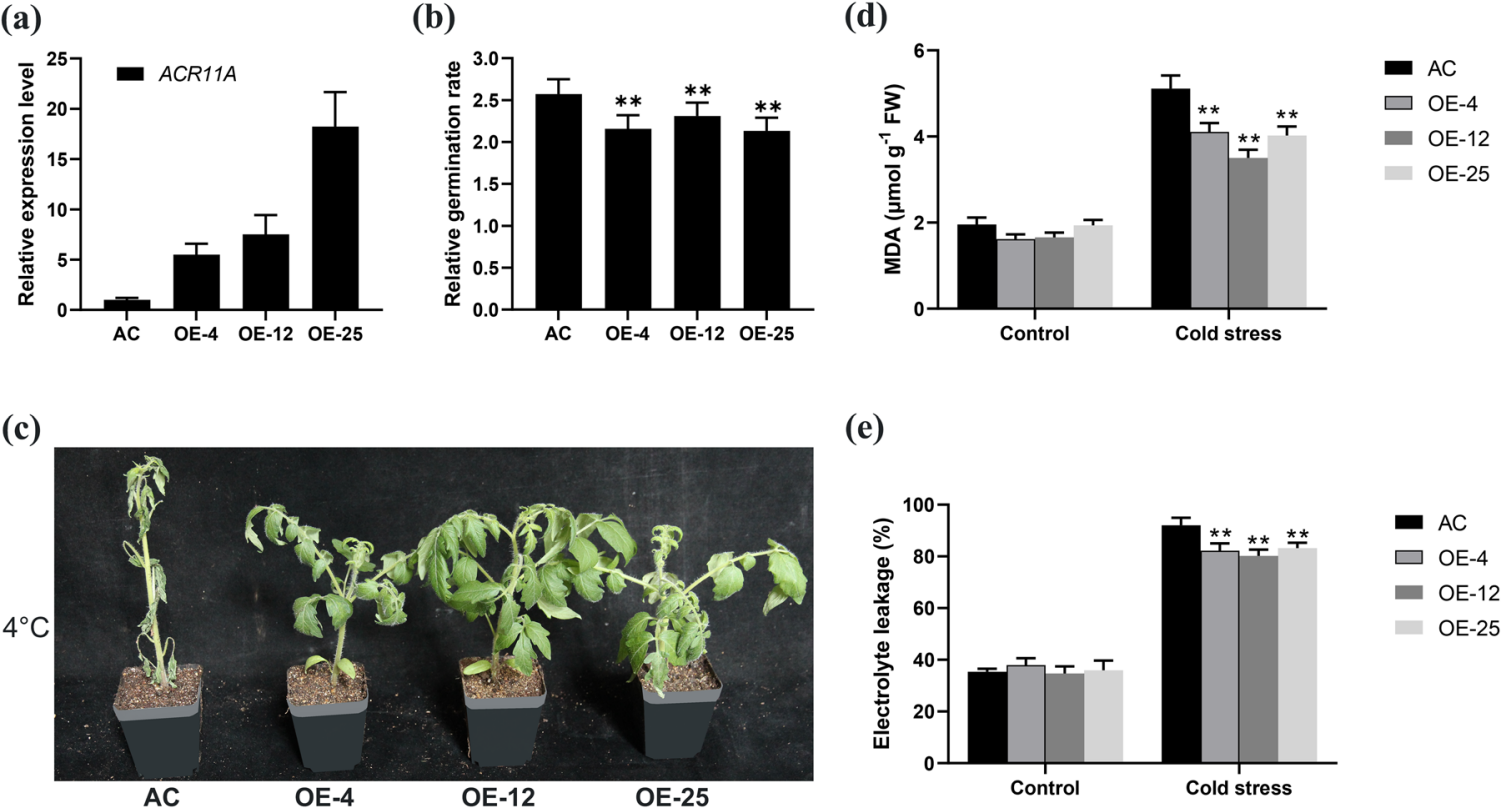
Overexpression of *SlACR11A* improved the cold tolerance of tomato. **a** Relative expression of *SlACR11A* in transgenic lines and wild-type (AC) plants. The expression levels were determined using qRT-PCR. The expression levels of the control (AC) were set to a value of 1. **b** Relative germination indices (rGIs) for transgenic lines and AC. To calculate the rGI, seeds from transgenic lines expressing abnormal levels of *SlACR11A* and AC were germinated at 25 °C and 15 °C for 7 days. The rGI was calculated using the germination index at 15 °C relative to 25 °C. All comparative germination assays involved three biological replicates. Approximately 100 seeds of each line were used for each replicate. **c** Phenotypes of transgenic and AC seedlings subjected to cold stress. Four-week-old T_2_ transgenic seedlings overexpressing *SlACR11A* (OE-4, OE-12, and OE-25) and AC seedlings were grown at 4 °C for 8 days during the summer. Comparison of MDA content (**d**) and relative electrolyte leakage (**e**) in the leaves of transgenic lines overexpressing *SlACR11A* and AC grown under cold and optimal conditions (control). Three biological replicates were analyzed for each line. Each replicate involved leaves of three seedlings. The error bars indicate the SEs. The asterisks indicate statistically significant differences relative to the wild type grown under the same conditions according to Student’s *t* test (*P < 0.05; **P < 0.01)

To investigate the biological functions of *SlACR11A* in tomato, we transformed AC with the 35 S:*SlACR11A* construct. In total, 24 stable transgenic lines were obtained. Three independent overexpression lines, OE-4, OE-12, and OE-25, were chosen for further characterization (Fig. 4a).

Four-week-old T_2_ and AC seedlings were chosen and tested for cold tolerance by growing them at 4 °C. After 8 days of cold stress, the AC seedlings were more severely wilted than the *SlACR11A* overexpression seedlings (Fig. 4c). Under optimal growth conditions, there were no differences in the levels of MDA or relative electrolyte leakage in the transgenic lines overexpressing *SlACR11A* and AC (Fig. 4d, e). After 8 days of cold treatment at 4 °C, the levels of MDA and relative electrolyte leakage increased in both transgenic plants that overexpressed *SlACR11A* and AC, but the levels were significantly lower in the *SlACR11A*-overexpression lines than in AC (Fig. 4d, e). These results indicate that *SlACR11A* can alleviate the damage to cellular membranes that occurs during cold stress. Cold stress also significantly affects seed germination. To evaluate cold tolerance, we compiled a relative germination index (rGI) at 15 °C relative to 25 °C; a lower rGI value indicates greater cold tolerance. After 7 days, the rGI of the lines that overexpressed *SlACR11A* was significantly lower than that of the control line (AC) (Fig. 4b). These data provide evidence that *SlACR11A* promotes cold tolerance during seed germination. Thus, we concluded that *SlACR11A* promotes cold tolerance during both seed germination and seedling growth by protecting cell membranes in tomato.

### Overexpression of *PP2-1* and *ACR11A* decreased the cold tolerance of tomato

**Fig. 5:**
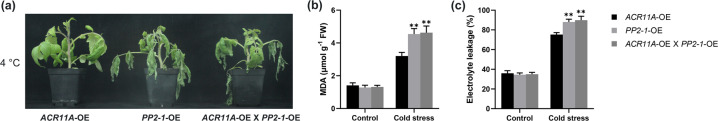
Overexpression of both *PP2-1* and *ACR11A* decreased the cold tolerance of tomato. **a** Phenotypes of *ACR11A* overexpression, *PP2-1* overexpression, and double overexpression seedlings subjected to cold stress. Four-week-old T_2_ transgenic seedlings were grown at 4 °C for 5 days. Comparison of MDA content (**b**) and relative electrolyte leakage (**c**) in the leaves of *ACR11A* overexpression, *PP2-1* overexpression, and double overexpression lines grown under cold and optimal conditions (control). Stable *ACR11A* overexpression, *PP2-1* overexpression, and three independent double overexpression lines were used for MDA and relative electrolyte leakage analysis. Three biological replicates were analyzed for each line. Each replicate was composed of leaves of three different seedlings. The error bars indicate the SEs. The asterisks indicate statistically significant differences relative to the appropriate control grown under the same conditions according to Student’s *t* test (*P < 0.05; **P < 0.01)

Based on our results, we conclude that *ACR11A* promotes the cold tolerance of tomato and that *PP2-1* attenuates cold tolerance. To study genetic interactions between *PP2-1* and *ACR11A*, we crossed lines overexpressing *PP2-1* and *ACR11A*. Four-week-old T_2_ seedlings that overexpressed *PP2-1*, *ACR11A*, or both *PP2-1* and *ACR11A* were tested for cold tolerance at 4 °C. After 5 days of treatment, the *PP2-1*-overexpression and double overexpression lines were severely wilted. In contrast, wilting occurred much less for the *ACR11A* overexpression plants (Fig. 5a). Moreover, we quantified the amounts of MDA and relative electrolyte leakage in the cold-treated and control plants and found similar physiological responses in the *PP2-1*-overexpressing lines and the double overexpression lines after 5 days of cold stress (Fig. 5b, c). Our data indicate that cold tolerance decreased in tomato plants that overexpressed both *PP2-1* and *ACR11A*.

### Favorable allele of *SlACR11A* in cold tolerance

**Fig. 6:**
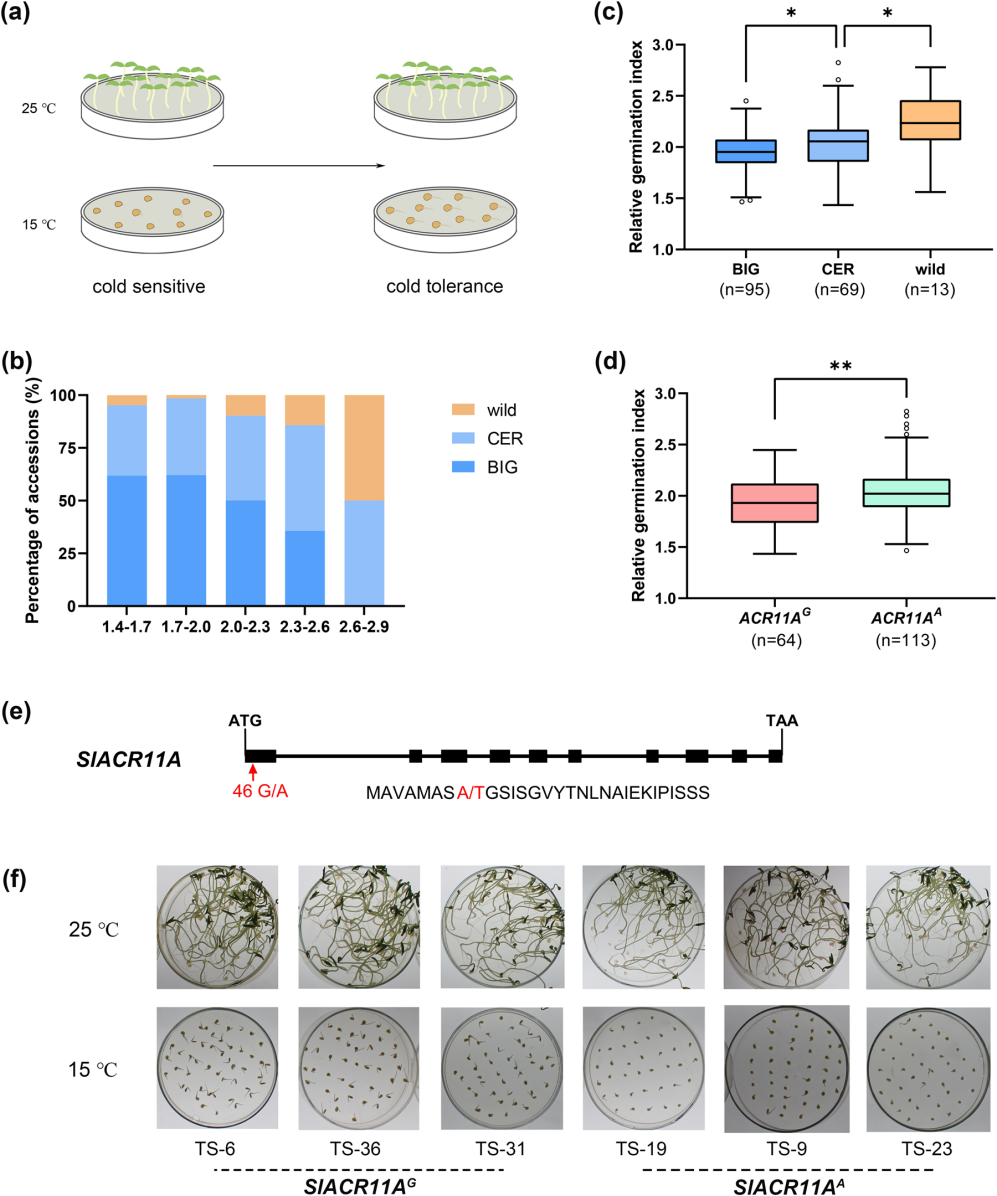
Distribution of *SlACR11A* alleles in tomato. **a** Diagram of the seed germination experiment. Germination at 15 °C was used to distinguish between cold-sensitive and cold-tolerant accessions. A temperate of 25 °C was considered an optimal condition (control) for seed germination. **b** Percentage of wild, CER, and BIG accessions with particular relative germination indices (rGI). **c** Variation in rGIs among the wild, CER, and BIG accessions. **d** Distribution of rGIs for 177 tomato accessions harboring either the *SlACR11A*^*G*^ or *SlACR11A*^*A*^ allele. For **b**, **c**, and **d**, seeds were germinated at either 25 °C or 15 °C for 7 days. The rGI was calculated using the germination index at 15 °C relative to 25 °C. Approximately 100 seeds of each accession were used in each germination assay. *P*-values were calculated using two-tailed Student’s *t* tests. **e** Gene structure of *SlACR11A*. The G/A SNP in the first exon causes an Ala-16 (A) to Thr (T) substitution. The boxes and lines represent exons and introns, respectively. **f** Seed germination of cold-tolerant and cold-sensitive accessions. Three accessions harbored the *SlACR11A*^*G*^ allele, and three accessions harbored the *SlACR11A*^*A*^ allele. Approximately 50 seeds of each accession were used in each germination assay, which was conducted at either 25 °C or 15 °C for 15 days

To learn more about genes that promote the cold tolerance of tomato, we used a population comprising 177 tomato accessions that included 13 wild accessions (1 *Solanum cheesmaniae*, 1 *Solanum galapagense*, and 11 *Solanum pimpinellifolium*), 69 domesticated accessions of *Solanum lycopersicum* var. *cerasiforme* (CER), and 95 improved accessions of *S. lycopersicum* (BIG) (Fig. 6a, f and Table S3). Among these 177 tomato accessions, the rGI ranged from 1.434 to 2.823. Thus, these tomato accessions present a spectrum of responses that range from cold tolerance to cold sensitivity (Table S3). The average rGI of accessions of wild species, CER and BIG were 2.234, 2.031, and 1.952, respectively. Thus, the rGI appears to gradually decrease from wild species to CER to BIG (Fig. 6c). In addition, as the rGI decreased, the percentage of wild tomato accessions gradually decreased, and the proportion of BIG tomato accessions increased. Indeed, the majority of accessions with low rGIs were BIG tomato accessions (Fig. 6b).

To identify genes regulating the cold tolerance of tomato, we analyzed the data generated by the tomato resequencing project from the tomato accessions whose degree of cold tolerance varied. We identified two alleles with a single nucleotide polymorphisms (SNP) in the first exon of *SlACR11A* changing Ala-16 to a Thr residue (Fig. 6e). The average rGI of the *SlACR11A*^*G*^ accessions was 1.930 (*n* = *64*); the average rGI of the *SlACR11A*^*A*^ accessions was 2.045 (*n* = *113*) (Fig. 6d, f and Table S3). These data indicate that the *SlACR11A*^*G*^ allele may afford cold tolerance and that the *SlACR11A*^*A*^ allele may afford cold sensitivity. Ala-16 is located within a nonconserved domain. Thus, relative to other members of the ACR protein family ACR11A may provide a unique function.

## Discussion

Identification of cold-tolerant germplasm and key genes involved in cold tolerance is critical for the genetic improvement of tomato. In this research, we elucidated a novel molecular mechanism in tomato involving an *ACR11A*-*PP2-1* module that regulates cold tolerance by preventing damage to cell membranes and identified a series of cold-tolerant and cold-sensitive tomato accessions, providing a theoretical basis and enriched germplasm for breeding.

Our data indicate that the expression of *ShPP2-1* from *S. habrochaites* increased in response to cold stress and that this gene negatively regulates cold tolerance in *S. habrochaites*. In this study, we analyzed our previously reported transcriptome data from cold-tolerant and cold-sensitive genotypes that had been subjected to cold stress^26^. We found that the expression of an F-box gene, *PP2-1*, was strongly induced by cold stress in a cold-tolerant tomato accession (LA1777) relative to a cold-sensitive tomato accession (AC) (Fig. 1a). We studied its contribution to cold tolerance and found that the overexpression of *ShPP2-1* in cultivated tomato (AC) reduced cold tolerance by intensifying the damage to cell membranes when tomato plants experience cold stress (Fig. 2). Generally, genes whose expression is upregulated by cold stress promote cold tolerance^28,29^. We believe that the response to *PP2-1* expression might differ between wild species (*S. habrochaites* accession LA1777) and cultivated tomato (AC). In the wild species (*S. habrochaites* accession LA1777), upregulation of *PP2-1* expression might downregulate the expression of a downstream gene that promotes cold tolerance. This response to *PP2-1* expression may not occur in cultivated tomato (AC), but this needs to be tested. Overall, cold stress induces a cold-tolerant phenotype for a wild tomato species (*S. habrochaites* accession LA1777).

To our knowledge, this report provides the first demonstration that SlACR11A is a novel PP2-1-interacting protein that promotes cold tolerance by preventing damage to cell membranes in tomato. To gain mechanistic insight into the role of *ShPP2-1* in cold tolerance, we conducted a yeast two-hybrid assay and found that PP2-1 can interact with ACR11A (Fig. 3). Consistent with the idea that these interactions are biologically meaningful, we found that overexpressing *SlACR11A* enhanced cold tolerance during seed germination and seedling growth (Fig. 4). Additionally, we quantified MDA and relative electrolyte leakage levels. We found that the overexpression of *SlACR11A* can alleviate the damage to cellular membranes during cold stress (Fig. 4d, e). Based on amino acid sequence similarities, we identified 14 genes that are paralogous to *SlACR11A* in tomato (Fig. S1); none of these genes have been studied previously. In contrast to tomato, there are 12 members of the Arabidopsis *ACR* gene family^23,24^. Only *AtACR11*—the ortholog of *SlACR11A*—was functionally characterized (Fig. S1). However, *AtACR11* has been reported to regulate disease resistance by affecting the accumulation of ROS and SA^25^.

Although PP2-1 and ACR11A interact in vitro and in vivo, the overexpression of only *ACR11A* and only *PP2-1* yielded distinct cold tolerance phenotypes (Figs. 2, 4, and 5). To obtain additional mechanistic insight, we overexpressed both *PP2-1* and *ACR11A* in tomato. We found that the cold tolerance phenotype of the double overexpression line was similar to that of the *PP2-1*-overexpression line (Fig. 5). Our data indicate that the overexpression of both *PP2-1* and *ACR11A* decreased the cold tolerance of tomato. Thus, the cold tolerance of the double overexpression line was suppressed by elevated levels of *PP2-1* expression. Thus, elevated levels of *PP2-1* expression may restrict the utilization of germplasm that expresses high levels of *ACR11A*. To avoid this problem, the expression level of *PP2-1* should be reduced before cold-tolerant varieties that express elevated levels of *ACR11A* are used for breeding. Thus, we should take advantage of the diversity of tomato germplasm resources and select the cold-tolerant germplasm that exhibits increased expression levels of *ACR11A* and reduced expression levels of *PP2-1* to accelerate the breeding process.

To learn more about resources and genes that can be used to promote the cold tolerance of tomato, we analyzed cold tolerance using a seed germination assay with a population that was subjected to severe cold stress (Fig. 6a). To exclude the possibility that differences in seed germination were responsible for the differences in cold tolerance, we calculated a relative germination index (rGI). We observed that cold tolerance is highly variable in the population (Table S3). We found that BIG germplasms appeared to be more cold tolerant than did wild tomato germplasms during germination (Fig. 6b, c). The changes in the proportions of these groups as rGIs decreased suggest that cold tolerance during germination might have been selected during tomato domestication and improvement. This is somewhat surprising but might be reasonable because fast germination in cold conditions of the open field promotes survival. Furthermore, we found that the *SlACR11A*^*G*^ allele is prevalent in cold-tolerant accessions and that the *SlACR11A*^*A*^ allele is prevalent in cold-sensitive accessions (Fig. 6d). Tomato originated in the Andes region of South America and was brought to Europe in the sixteenth century^30^. Tomato is widely bred for cultivation in temperate regions and has developed into a major horticulture crop species that is grown worldwide. During the process of tomato domestication, particular alleles or genes linked either to environmental stress or to desirable traits may have been gained or lost from the wild ancestors^31–34^. For example, the *FIS1* locus that contributes to fruit firmness was positively selected during tomato domestication^35^. On the other hand, the *SlHAK20* locus, which contributes to the transport of both Na^+^ and K^+^ during salt stress, was lost during domestication^36^. *SlACR11A*^*G*^ was probably selected as a gain-of-function allele that promotes cold tolerance for the cultivation of tomato in a particular climate. Therefore, the *SlACR11A*^*G*^ allele is highly valuable for the breeding of enhanced cold tolerance and should be studied.

In summary, we found that *ShPP2-1* is a novel gene whose expression is induced by cold and that negatively regulates cold tolerance by intensifying cell membrane damage when plants experience cold stress. In addition, we demonstrated that ACR11A interacts with ShPP2-1 and promotes the cold tolerance of tomato. We found that overexpression of both *PP2-1* and *ACR11A* decreased the cold tolerance of tomato. Furthermore, our analyses of a population indicate that *SlACR11A*^*G*^ is a naturally occurring allele that is significantly enriched in cold-tolerant tomato plants and that the *SlACR11A*^*A*^ allele is significantly distributed in cold-sensitive tomato accessions. These data provide evidence that *SlACR11A*^*G*^ might play a role in cold tolerance. Therefore, our study demonstrates that the combination of low expression levels of *PP2-1* and high expression levels of *ACR11A* can serve as direct targets for both genetic engineering and selection for enhanced cold tolerance of tomato. These findings provide essential information for breeding.

## Materials and methods

### Plant materials and growth conditions

We analyzed a total of 177 tomato accessions from a collection that was published previously^31^ and that included 1 *S. cheesmaniae*, 1 *S. galapagense*, 11 *S. pimpinellifolium*, 69 *S. lycopersicum* var. *cerasiforme*, and 95 *S. lycopersicum* accessions (Table S3). Seeds that were used for germination assays were extracted as previously described^37^. Wild tomato (*S. habrochaites*) accession LA1777 and cultivated tomato (*S. lycopersicum* cv. Ailsa Craig (AC)) were used for the quantification of gene expression. AC was selected for genetic transformation. Transgenic plants from the T_2_ generation and AC plants expressing abnormal levels of *ShPP2-1* and *SlACR11A* were tested for their tolerance to cold stress. All of the plants were grown under a photoperiod consisting of 16 h of light and 8 h of darkness in a greenhouse at 25 ± 2 °C and a relative humidity of 70%.

### Abiotic stress tolerance assays

The cold-tolerant *S. habrochaites* accession LA1777 and cold-sensitive *S. lycopersicum* cv. AC were used to study the expression of *PP2-1*. Four-week-old seedlings were subjected to abiotic stress treatments, namely, cold, drought, salt, and heat stress treatments. The methods corresponding to these abiotic stress treatments were described previously^38^. After different time points for the different treatments, the third fully expanded leaf from the growing point was collected for the isolation of RNA.

For cold stress tolerance experiments with transgenic plants, 4-week-old seedlings of T_2_ transgenic lines and wild-type (AC) plants were chosen and placed randomly in a growth chamber at 4 °C, under 70% relative humidity and under a photoperiod consisting of 16 h of light and 8 h of darkness. The seedlings of the control group were grown in a separate growth chamber with identical conditions except that the temperature was 25 °C. For transgenic lines expressing abnormal levels of *PP2-1* (Fig. 2) and *ACR11A* (Fig. 4), seedlings were grown at low temperatures for either 6 days (in the winter) or 8 days (in the summer). Transgenic seedlings that overexpressed both *PP2-1* and *ACR11A* (Fig. 5) were treated for 5 days. After the cold stress treatment, the third fully expanded leaf from the top of each seedling was collected, frozen in liquid nitrogen, stored at −80 °C, and subsequently used for malondialdehyde (MDA) measurements.

### Quantitative real-time PCR analysis

Total RNA was extracted using TRIzol reagent (Aidlab, China). First-strand cDNA was synthesized using a HiScript II 1st Strand cDNA Synthesis Kit (+gDNA wiper) (Vazyme, China), as recommended by the manufacturer. *PP2-1* and *ACR11A* expression levels were analyzed using quantitative real-time PCR (qRT-PCR), which was conducted using a QuantStudio^TM^ 6 Flex System (ABI, USA). The tomato actin gene (Solyc11g005330) was used as an internal control. The sequences of all the primers that were used for qRT-PCR are listed in Table S1. For each biological replicate, the relative expression was quantified using three technical replicates and calculated using the 2^-ΔΔCT^ method^39^.

### Generation of transgenic tomato plants

For overexpression constructs, the complete open reading frame (ORF) of *PP2-1* was amplified from LA1777 using PP2-1-OE primers (Table S1). The full-length ORF sequence of *ACR11A* was amplified from AC using ACR11A-OE primers (Table S1). Both of these constructs were cloned into pHELLSGATE8 vectors, which use the *Cauliflower mosaic virus* (CaMV) 35 promoter to drive expression, using a CloneExpress^®^ II One-Step Cloning Kit (Vazyme, China). To generate RNAi constructs, a 110-bp fragment from the *PP2-1* gene was amplified from cDNA that was prepared from AC using the PP2-1-Ri primers (Table S1) and cloned into pHGRV using Clonase BP (Invitrogen, USA). AC was transformed with all of the recombinant plasmids using *Agrobacterium tumefaciens*-mediated transformation. The cotyledons of tomato were used for transformation^40^. Positive transgenic plants were screened via PCR with a CaMV 35 S promoter primer and a gene-specific primer. After PCR and qRT-PCR analyses, several independent homozygous transgenic lines expressing abnormal levels of *PP2-1* (OE-6, OE-8, Ri-2, and Ri-13) and *ACR11A* (OE-4, OE-12, and OE-25) were selected for further analysis. To generate double-overexpression plants, plants that overexpressed *ACR11A* and *PP2-1* were crossed. Plants that overexpressed both genes were selected from the progeny.

### Germination experiments

For the 177 tomato accessions and the transgenic lines expressing abnormal levels of *SlACR11A*, approximately 100 seeds were germinated in 9 cm petri dishes with two layers of filter paper that were moistened with distilled water and incubated in growth chambers in the dark at either 25 °C or 15 °C. To quantify germination, seeds that germinated were counted each day. After 7 days, a germination index (GI) was calculated using the following formula: *GI* = *(Σ T*_*i*_ *N*_*i*_*) / S*, where *Ti* represents the time (in days) between seed imbibition and germination, *Ni* represents the number of seeds germinated each day, and *i* and *S* represent the total number of seeds germinated^12^. To further correct for differences in seed vigor among different accessions, a relative germination index (*rGI*) was calculated using the following formula: *rGI* = *GI (15* °C*) / GI (25* °C*)*, where *GI (15* °C*)* represents the germination index at 15 °C and where *GI (25* °C*)* represents the germination index at 25 °C. All comparative germination assays involved the use of three biological replicates.

### Measurements of relative electrolyte leakage and malondialdehyde levels

Relative electrolyte leakage and malondialdehyde (MDA) levels were used to evaluate damage to cellular membranes^27^. The third fully expanded leaf from the top of both cold-stressed and control transgenic and wild-type plants were collected. The relative electrolyte leakage and MDA levels were determined as previously described^29^. Three biological replicates were analyzed for each sample.

### Yeast two-hybrid assays

The CDSs of *ShPP2-1* and *SlACR11A* were amplified from LA1777 and AC using sequence-specific primers (Table S1) and incorporated into pGBKT7 and pGADT7 vectors (Clontech, USA), respectively. According to the manufacturer, the recombinant plasmids pGBKT7-53 and pGADT7-RecT (positive control), pGBKT7-PP2-1 and pGADT7-ACR11A, pGBKT7-PP2-1 and pGADT7, pGBKT7 and pGADT7- ACR11A, and pGBKT7-Lam and pGADT7-RecT (negative control) were introduced into the *Saccharomyces cerevisiae* strain AH109. To evaluate the interactions, the transformants were grown on SD/-Trp/-Leu and SD/-Trp/-Leu/-Ade/-His media.

### Bimolecular fluorescence complementation assays

The full-length CDSs of *ShPP2-1* and *SlACR11A* without stop codons were incorporated into pUC-SPYNE and pUC-SPYCE, respectively. Tobacco protoplasts were bombarded with either pUC-SPYNE-PP2-1 and pUC-SPYCE-ACR11A or pUC-SPYNE-PP2-1 and pUC-SPYCE (negative control). Tobacco protoplasts were isolated and transformed as described previously^41^. The protoplasts were transformed with pGhd7-CFP, which was used as a nuclear marker. After incubating the protoplasts at 24 °C for 18 h, YFP fluorescence was imaged using a Leica TCS SP8 confocal laser scanning microscope (Leica, Germany) and Leica Application Suite X 3.7.1.21655 software.

### *SlACR11A* genotyping, evolution, and phylogenetic analysis

Polymorphic sequences within the *SlACR11A* gene from 177 tomato accessions were downloaded from the Sol Genomics Network (https://solgenomics.net/)^31^. Detailed information on the accessions is provided in Table S3. Boxplots with evolution and swarm layouts were plotted using GraphPad Prism. For the phylogenetic analysis of the ACR protein family, the amino acid sequences of ACR proteins from Arabidopsis and tomato were downloaded from TAIR (http://www.arabidopsis.org/) and the Sol Genomics Network (https://solgenomics.net/), respectively. Full-length amino acid sequences of tomato ACR1 to ACR13 and Arabidopsis ACR1 to ACR12 were aligned using ClustalW2. To construct a phylogenetic tree, the neighbor-joining algorithm was used, and bootstrap analysis was performed using 2000 replicates. Evolutionary analysis was conducted via MEGA 7^42^.

### Statistical analyses

Statistical analyses were performed using GraphPad Prism software. Statistically significant differences between two groups were determined using a two-tailed Student’s *t* test. *P* < 0.05 (*) and *P* < 0.01 (**) were considered to be statistically significant.

### Accession numbers

The sequence data for the following genes (accession numbers) were downloaded from the Sol Genomics Network (http://solgenomics.net/):

*ShPP2-1* (Solyc05g055870); *SlACR1* (Solyc02g092150); *SlACR2* (Solyc06g011520); *SlACR3* (Solyc06g059800); *SlACR4* (Solyc01g010150); *SlACR5* (Solyc05g011920); *SlACR6* (Solyc02g094300); *SlACR7* (Solyc08g007540); *SlACR8* (Solyc08g079650); *SlACR9* (Solyc05g054620); *SlACR10* (Solyc09g008350); *SlACR11A* (Solyc03g117890); *SlACR11B* (Solyc06g068670); *SlACR12* (Solyc11g006970); and *SlACR13* (Solyc01g010000).

## Supplementary information

Supplementary Figure S1

Supplementary Tables S1-S2

Supplementary Table S3
