# Supplementary material for: Interactions between ShPP2-1, an F-box family gene, and ACR11A regulate cold tolerance of tomato
Source: Hortic Res. 2021 Jul 1;8:148. doi: 10.1038/s41438-021-00582-3 (PMC8245493; doi:10.1038/s41438-021-00582-3)
Supplement: Supplementary file 1 — Supplementary Figure S1 [file 41438_2021_582_MOESM1_ESM.pdf]

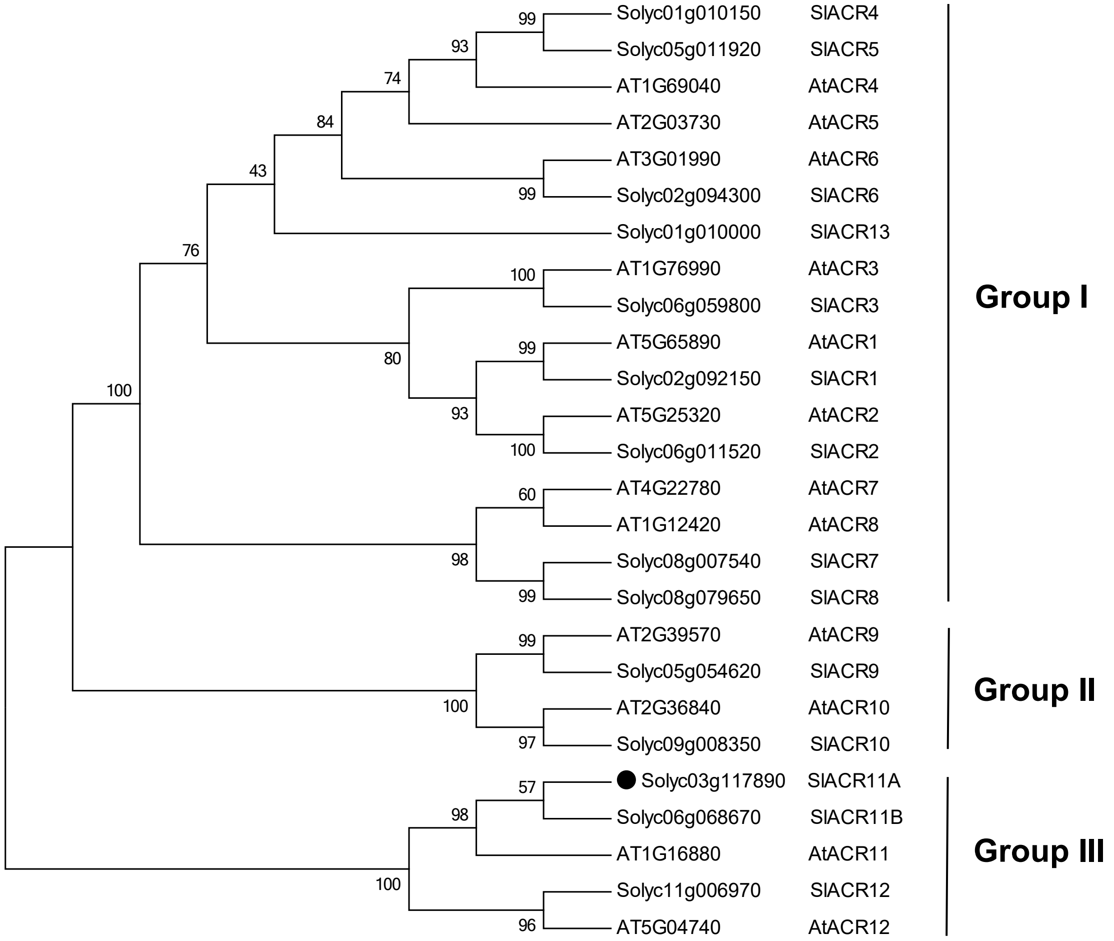

Fig. S1 Sequence analysis of the tomato ACR family members.

Phylogenetic relationships among ACR proteins in tomato and Arabidopsis. The full-length amino acid sequences of tomato ACR1 to ACR13 and Arabidopsis ACR1 to ACR12 were aligned using ClustalW2. The phylogenetic tree was constructed using the neighbor-joining algorithm. Evolutionary analyses were conducted via MEGA 7. Twelve Arabidopsis ACR proteins and 14 tomato ACR proteins were divided into 3 groups.
