# Supplementary material for: Interactions between ShPP2-1, an F-box family gene, and ACR11A regulate cold tolerance of tomato
Source: Hortic Res. 2021 Jul 1;8:148. doi: 10.1038/s41438-021-00582-3 (PMC8245493; doi:10.1038/s41438-021-00582-3)
Supplement: Supplementary file 2 — Supplementary Tables S1-S2 [file 41438_2021_582_MOESM2_ESM.pdf]

Table S1. List of primers used in this study

Abbreviation	Primer sequence (5′-3′)	Description
ACR11A-OE-FW	CATTTGGAGAGGACACGCTCGAGATGGCTGTGGCTATGGCTTC	Transgenic vector construction
ACR11A-OE-RV	TCTCATTAAAGCAGGACTCTAGATTAGAAGCTTGACTCGTTTGTTGTT	
PP2-1-OE-FW	CATTTGGAGAGGACACGCTCGAGGCTCATTTATGTTCTTACCCATTACCA	
PP2-1-OE-RV	TCTCATTAAAGCAGGACTCTAGACCAAATGAACCTTTTCAGTATCAAGTT	
PP2-1-Ri-FW	AAAAAGCAGGCTGGAAGGTTGCATAGCCAACGTACTAT	
PP2-1-Ri-RV	AGAAAGCTGGGTCCCAAACGGCGTCAGATTCAC	
attB1	GGGGACAAGTTTGTACAAAAAAGCAGGCT	
attB2	GGGGACCACTTTGTACAAGAAAGCTGGGT	
35S	ACGCACAATCCCACTATCCTTC	CaMV 35S promoter
Qactin-Fw	GTCCTCTTCCAGCCATCCA	Q-PCR standard control
Qactin-Rv	ACCACTGAGCACAATGTTACCG	
ACR11A-Q-FW	TCTTCAGAGGTTCTTTTGGTTTGG	Q-PCR
ACR11A-Q-RV	TTTGGGCGTTGGGACAGC	
PP2-1-Q-FW	GTTTTTGCCGTCGGATTACC	
PP2-1-Q-RV	CCGTCGATGAAGAAAGGATGA	
PP2-1-BD-FW	ATGGCCATGGAGGCCGAATTCATGGCGGTGGAAATTGGAC	Yeast two-hybrid
PP2-1-BD-RV	CCGCTGCAGGTCGACGGATCCTTACCCTTCCTTAGGCCTGATTT	
ACR11A-AD-FW	GCCATGGAGGCCAGTGAATTCATGGCTGTGGCTATGGCTTC	
ACR11A-AD-RV	CAGCTCGAGCTCGATGGATCCTTAGAAGCTTGACTCGTTTGTTGTT	
PP2-1-pUC-SPYNE-FW	TGGCGCGCCACTAGTGGATCCATGGCGGTGGAAATTGGAC	Bimolecular fluorescence complementation
PP2-1-pUC-SPYNE-RV	CCCGGGAGCGGTACCCTCGAGCCCTTCCTTAGGCCTGATTT	
ACR11A-pUC-SPYCE-FW	TGGCGCGCCACTAGTGGATCCATGGCTGTGGCTATGGCTTC	
ACR11A-pUC-SPYCE-RV	CCCGGGAGCGGTACCCTCGAGGAAGCTTGACTCGTTTGTTGTT	

Table S2. List of genes identified using a yeast two-hybrid assay.

Gene ID	Annotation
Solyc01g066430	Ring H2 finger protein
Solyc01g108630	Nitrite reductase
Solyc01g111450	Proteasome subunit alpha type
Solyc02g067460	Porin/voltage-dependent anion-selective channel protein
Solyc02g089250	Pollen Ole e 1 allergen and extensin
**Solyc03g117890**	Unknown protein DS12 from 2D-PAGE of leaf, chloroplastic
Solyc03g121180	GDSL esterase/lipase At5g22810
Solyc04g011430	Ubiquitin-conjugating enzyme 13 E2
Solyc06g005060	Elongation factor 1-alpha
Solyc06g009970	Elongation factor 1-alpha
Solyc06g071430	T17H3.1 protein (Fragment)
Solyc07g032090	Transcription initiation factor IIF subunit beta
Solyc09g008800	60S ribosomal protein L24
Solyc12g005860	3-isopropylmalate dehydratase large subunit
